# Yearly Variations in GCM Concentrations in Female Mountain Hares (*Lepus timidus*) and the Effect of Pregnancy

**DOI:** 10.3390/ani11092710

**Published:** 2021-09-16

**Authors:** Maik Rehnus, Rupert Palme

**Affiliations:** 1 Swiss Federal Research Institute for Forest, Snow and Landscape Research WSL, Zürcherstrasse 111, 8903 Birmensdorf, Switzerland; 2 Department of Biomedical Sciences, University of Veterinary Medicine, Veterinärplatz 1, 1210 Vienna, Austria; Rupert.Palme@vetmeduni.ac.at

**Keywords:** Alps, noninvasive genetic sampling, pellets, stress hormones, reproduction, season, weather

## Abstract

**Simple Summary:**

The measurement of stress hormones has become a widely used and effective tool for evaluating adrenocortical activity in animals. However, to correctly interpret stress measurements, the potential sampling bias resulting from an oversampling of individuals in different states of pregnancy has rarely been investigated. We found significant yearly variations in states of pregnancy, which is related to the conditions of the females due to the snow cover duration before and at the start of the reproductive period. These results are important for improving the interpretation of stress hormone concentrations in free-ranging populations during the breeding and reproductive periods.

**Abstract:**

The measurement of glucocorticoid metabolites (GCMs) in faeces has become a widely used and effective tool for evaluating the amount of stress experienced by animals. However, the potential sampling bias resulting from an oversampling of individuals in different states of pregnancy has rarely been investigated. In this study, we validate a noninvasive method for measuring gestagen metabolites in female mountain hares (*Lepus timidus*) under controlled conditions. We also measured the concentration of gestagen metabolites of females in a free-ranging population during the early breeding and post-breeding periods from 2014 to 2019. We found significant yearly variations in gestagen metabolites, which were related to the condition of the females due to the snow cover duration before and at the start of the reproduction period. GCMs were significantly influenced by the gestagen metabolite levels. These results are important for improving the interpretation of GCM concentrations in free-ranging populations during the breeding and reproductive periods.

## 1. Introduction

The measurement of glucocorticoid metabolites (GCMs) has become a widely used and effective tool for evaluating adrenocortical activity in animals [1,2]. Faecal GCM measurements can be easily obtained without any need to handle the animal, rendering the sampling process almost feedback-free and, therefore appropriate for evaluating stress in free-ranging wild animals [3]. However, GCM excretion may depend on the reproductive state of the animal responsible for the collected sample [4]. To correctly interpret GCM measurements [2], it is important to clarify this relationship.

Our model species, the mountain hare (*Lepus timidus*), is a perfect species for testing the influence of different reproductive states on GCM excretion, because GCM and noninvasive genetic sampling (NIGS) methods have recently been developed specifically for this purpose [5,6,7,8,9]. The mountain hare is an elusive species that is nocturnally active, has no sexual dimorphism, and is sensitive to disturbances [9,10,11]. It is a nonterritorial species, and individual home ranges show considerable overlap [12]. Mountain hare females typically have two to three litters during a single reproductive period in the Alps, which regularly starts in April and ends in August [13]. The reproductive success of mountain hares is threatened by climate change and by stress due to human recreational activities [9,14,15]. To understand how hare reproduction is affected by these stressors, we need to better understand how reproduction affects GCM excretion. To this end, a noninvasive method for endocrine monitoring of pregnancy has to be developed.

In this study, we validate a noninvasive method for measuring gestagen metabolites in female mountain hares under controlled and field conditions. First, we evaluated the suitability of enzyme immunoassays as indicators of the state of pregnancy. Secondly, we investigated the seasonal and yearly variations in gestagen metabolites from a collection of faeces in a free-ranging population over six consecutive years, 2014–2019, in the early breeding and the post-breeding periods and how the concentrations of gestagen metabolites influence the GCM levels. Finally, we discuss how this knowledge can be used to improve the interpretation of the results of faecal glucocorticoid metabolite measurements in free-ranging populations.

## 2. Materials and Methods

### 2.1. Noninvasive Method for Measuring States of Pregnancy

To establish noninvasive methods for the endocrine monitoring of pregnancy indicated by concentrations of gestagen metabolites in mountain hares, we obtained 81 faecal samples in the “Zoo am Meer” in Germany. We analysed them with a 5ß-pregnane-3α-ol-20-one enzyme immunoassay (EIA) to measure 20-oxopregnanes (20-OP) and a pregnanediol EIA to measure 20α-hydroxypregnanes (20α-OHP). The sampling was conducted from 2017 to 2018 during the early breeding period (March and April), the breeding period (May and June), and the post-breeding period (November–February). Young hares were born during the sampling period (April: *n* = 1, May: *n* = 1, and June: *n* = 4).

### 2.2. Fieldwork

The study area comprised 3.5 km^2^ and was situated along the Ofenpass in the Swiss National Park in South-eastern Switzerland (46°39′N, 10°11′E). The Swiss National Park is designated by the International Union for the Conservation of Nature [16] as a Category Ia nature reserve (strict nature reserve/wilderness area) and is closed to the public in the winter. It was therefore possible to study mountain hares under natural conditions without human disturbance.

The study area ranged in elevation from 1693 to 2587 m a.s.l. The habitats within the study area were delimited and classified according to the habitat categories of HABITALP, a standardised habitat classification project for protected areas in the Alps [17]. The study area encompassed seven main habitat types: meadows (29%; with diverse grasses, including *Nardus stricta*, *Festuca* sp., *Poa* sp., *Agrostis* sp., *Luzula* sp., and sedges); timber stands (24%); scree slopes (16%); storeyed stands (12%; mixed *Larix decidua*, *Pinus cembra*, *P. sylvestris*, *P. mugo* spp., and *Picea abies*); sapling stands (6%; dominated by *P. mugo* spp.); pole timber (5%); and mature stands (5%). Residual habitats covered 3% of the area. The climate in the Swiss National Park is continental, with mean January and July temperatures of –9 °C and 11 °C, respectively [18]. The mean monthly precipitation measured at 1970 m a.s.l. is 34 mm in January and 108 mm in July [18].

We collected fresh pellets over six consecutive years (2014–2019) in the early breeding (end of March until the first half of April) and in the post-breeding periods (October). The samples were collected both systematically and opportunistically, as described in detail by Reference [5]. Systematic sampling was conducted on 91 plots that were preselected on a 200-m-square grid; all hare pellets within each trial plot were collected during each visit. For the opportunistic sampling, we collected pellets as we moved from one systematic plot to the next. Only fresh faecal pellets were collected, because amplification success rates are significantly lower for pellets older than five days [5] and because GCM can be influenced by weather conditions [6]. Samples for the genetic analyses were collected and stored in separate plastic tubes without being touched by hand to minimise DNA contamination [19]. Samples for the GCM analyses were stored in plastic bags. After collection in the field, the samples were frozen and stored until they were analysed in the lab.

### 2.3. Genetic Analyses

We used nine nuclear microsatellites to identify individuals and assign them to the collected faeces samples: Lsa1, Lsa3 [20], Sat2, Sat5, Sat8, Sat12 [21], Sol30, Sol8 [22], and Sol33 [23]. Seven loci were analysed in R [24], and two (Sat2, Sat12) were scored qualitatively using a description of a phenotypic peak to find consensus genotypes for each replicated sample [25]. The sex of the pellet owner was determined using an assay developed by Reference [26]. The assay is only amplified in male individuals, as it amplifies part of the Y-chromosomal SRY [26]. A genotype is considered female if none of the three replicates are amplified at the SRY locus and male if at least one of the replicates is amplified. For the identification of unique genotypes, the ALLELEMATCH package in R was used, which considers genotyping errors and missing data during the assignment of individuals [27]. A unique individual is identified when the sample differs from all other samples at more than two loci, including the additional loci (Sat2 and Sat12). The DNA samples were genotyped by three independent replicates, and consensus homozygote genotypes were accepted if all three replicates were consistent. Consensus heterozygote genotypes were accepted if at least two replicates were consistent, and no more than two alleles were found across all three replicates [25].

### 2.4. GCM Analyses

Faecal GCMs were measured using an 11-oxoaetiocholanolone EIA, which has proven suitable (based on the results of a validation study including an ACTH challenge test) for evaluating the adrenocortical activity in mountain hares [6]. Every sample was dried and thoroughly homogenised. Afterwards, a portion (0.15 g) was mixed with 5 mL of methanol (80%), shaken (30 min), and centrifuged (2500× *g*; 15 min). An aliquot of the supernatant (after 1:10 dilution with an assay buffer) was then analysed in the 11-oxoaetiocholanolone EIA. All intra- and inter-assay coefficients of variation were below 12%, and the sensitivity of the method was 2 ng/g of faeces. The details of the extraction procedure and the EIA can be found elsewhere [6,28,29].

### 2.5. Pregnancy State Analyses

The same extraction procedure as for faecal GCM was applied to measure the pregnancy status of the individuals. Aliquots of the extract were analysed in a 5ß-pregnane-3α-ol-20-one EIA (measuring 20-oxopregnanes: 20-OP) and a pregnanediol EIA (measuring 20α-hydroxypregnanes: 20α-OHP). The details of the EIAs, including any cross-reactions, can be found elsewhere [30,31].

### 2.6. Statistical Analyses

All statistical tests were conducted using R 3.6.3 [24].

To investigate the seasonal differences in gestagen metabolites under controlled conditions, we used 81 samples in a linear model with EIA (20-OP and 20α-OHP) as the response variable and season (early breeding period, reproductive period, and post-breeding period) as the predictor variable.

**Figure 1:**
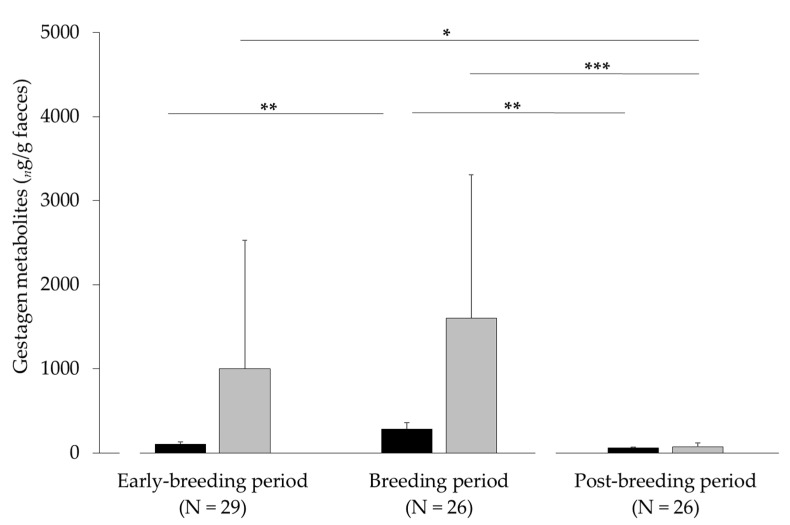
Concentrations (mean ± SD) of gestagen metabolites (black = 20-OP and grey = 20α-OHP) during different phases of the reproduction period of mountain hares from 2017 to 2018 (* *p* < 0.05, ** *p* < 0.01, and *** *p* < 0.001; the lower lines compare 20-OP and the upper two lines the 20α-OHP levels).

To investigate the variations in the reproduction activity of free-ranging female mountain hares over six years (2014–2019), we used 248 samples from 33 individuals (*n* = 138 from the early breeding period and *n* = 110 from the post-breeding period) and measured 20α-OHP based on the higher sensitivity and clearer distinction of the post-breeding period (Figure 1). The compositions of females with different pregnancy states were classified into four categories (≥3000 ng/g, 2000–2999 ng/g, 1000–1999 ng/g, and <1000 ng/g) and used to show the yearly variations in the pregnancy states of the females (Table 1). We used linear models with gestagen metabolites as the response variable, year, and season (early breeding period and post-reproductive period) as the predictor variables and individual ID as a random factor. In the investigation of variations in GCM concentrations, we used linear models with GCM as the response variable, year, season (early breeding period and post-breeding period), gestagen metabolites, and the interaction season gestagen metabolites as the predictor variables and individual ID as a random factor. We used the Shapiro–Wilk normality test to examine the distribution of data. If the variables were not normally distributed, they were transformed to meet the criteria of normal distribution.

## 3. Results

### 3.1. Noninvasive Method for Measuring States of Pregnancy

The seasonal differences in gestagen metabolites, with peaks during the breeding period, were significant for 20-OP (F_2,78_ = 6.32, *p* = 0.003) and 20α-OHP (F_2,78_ = 8.77, *p* < 0.001; Figure 1). 20α-OHP were present at higher concentrations and enabled a clearer distinction between the post-breeding period as compared to the early breeding and breeding periods (Figure 1).

### 3.2. Fieldwork

The composition of females with different states of pregnancy during the early breeding period varied among the years (Table 1). In 2014 and 2018, the years in which we found no females with the highest gestagen metabolite levels (≥3000 ng/g) during the early breeding period, we found a higher January–March snow height as compared to the other years. We found no clear pattern for the other variables (number of days with snowfall, average daily temperatures, and number of days with air frost; Table 1). In 2019, we found one female with the highest gestagen metabolites level (≥3000 ng/g) also during the post-breeding period.

**Figure 2:**
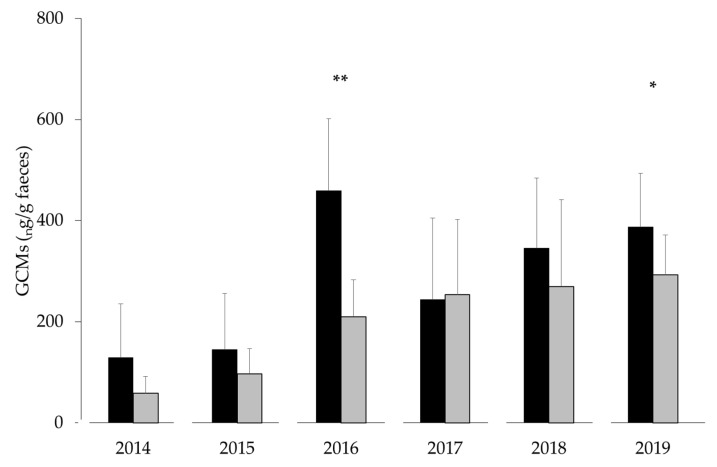
Fluctuations in the GCM concentrations (mean ± SD) of female mountain hares during the early breeding period (black) and the post-breeding period (grey) in the Swiss National Park from 2014 to 2019 (* *p* < 0.05 and ** *p* < 0.01).

The GCM concentrations varied significantly from year to year (F_5,207_ = 20.97, *p* < 0.001; Figure 2). We found that the GCMs were significantly influenced by gestagen metabolites (F_1,207_ = 5.42, *p* = 0.021), where the GCMs increased with the increasing concentrations of gestagen metabolites. For example, in 2016, the highest GCM concentrations were found during the early breeding period, which was when the most pregnant females were found in the study area (Table 1). The GCM concentration was also significantly influenced by the season (F_1,207_ = 25.64, *p* < 0.001), with higher GCM excretions occurring during the early breeding period as compared to the post-breeding period (Figure 2). The interaction season gestagen metabolites had no significant influence on the GCM concentrations (F_1,207_ = 0.68, *p* = 0.410).

## 4. Discussion

We validated a noninvasive method for measuring gestagen metabolites as indicators of different states of pregnancy in female mountain hares and showed variations in a population of free-ranging mountain hares over a period of six years. These results are important for understanding how the timing of the mountain hare reproduction period varies from year to year [32]. They also improve the interpretation of faecal glucocorticoid results when collecting “anonymous” faeces in free-ranging populations during reproductive periods.

The selected 20-OP and 20α-OHP EIAs [30,31], used here for the first time in the genus *Lepus*, exhibited the required suitability for measuring the gestagen metabolite concentrations in mountain hare faeces. However, the results of both EIAs differed significantly in the measured amounts and in distinction between the early breeding and the post-breeding periods. The 20α-OHP EIA proved better-suited as an indicator of the state of pregnancy (luteal phase/pregnancy) in mountain hares.

We found a high yearly variation in the compositions of females with different pregnancy states in the early breeding season in the Alps, which was in alignment with the observations of mountain hares in North-eastern Scotland [33]. Few females with the highest gestagen metabolites level (≥3000 ng/g) were observed during the early breeding period in our study, but the number of pregnant females could be expected to increase later in the year [33].

Year-to-year changes in the distribution of the pregnancy states throughout the year could be attributed to weather conditions. In particular, the snow cover in January–March affects the conditions for reproduction [33,34]. Snow limits access to the nutrient-rich food that is preferred by females in the spring when the reproductive period begins [35]. The temperature seems to play a secondary role in the state of pregnancy, a conclusion supported by observations of other mountain hare populations [36].

We would expect the pregnancy state of females during the early breeding period to have the most direct influence on the success of the first litters in April [10], because the pregnancy state influences the onset and length of the reproduction season. This is the case in mammals like snowshoe hares (*Lepus americanus*) and cottontail rabbits (*Sylvilagus floridanus*) [37,38,39]. It may be possible that the conditions for reproduction and the survival of the first litters in April may improve as the climate warms and the amount of snow in the Alps decreases [40,41], because more food is available earlier in the year. However, the mountain hare is a cold-adapted species and can therefore only move upward with higher temperatures; it will not find suitable environmental conditions at lower elevations [15]. Mountain hares have to follow the “green wave” up the mountain to find high-quality food until autumn [42].

Interestingly, we found one pregnant female with the highest gestagen metabolites level (≥3000 ng/g) in the post-breeding period at the beginning of October. This can be interpreted as another indicator of the flexibility of mountain hares with regards to their reproductive strategy [13].

The GCM concentrations in the females were influenced by year, season, and gestagen metabolites. The season affects the GCM concentrations due to the season-dependent energetic costs (which also vary from year to year). The early breeding season has a higher energetic cost due to the activation of catabolic processes associated with hypothalamic–pituitary–adrenal (HPA) axis activation, ovulation, and pregnancy [43,44], all of which lead to higher GCM concentrations in various mammal species [4]. Gestagen metabolites have a positive effect on GCMs, the strength of which depends on the pregnancy states of the females [4].

Interestingly, we found differences in the GCMs between the early breeding and post-breeding periods but no differences in the gestagen metabolites. This confirms the influence of other factors on the pregnancy state (like the predation risk) that are tightly correlated with maternal off-early breeding period stress profiles [45,46], individual differences [7], and the population cycle [39,47].

## 5. Conclusions

Our results showed how the evaluated enzyme immunoassays can be used as indicators of the state of pregnancy to improve the interpretation of the results of faecal glucocorticoid metabolites in free-ranging populations of mountain hares. We recommend that other GCM studies take into account the pregnancy state of females during the breeding and reproductive periods to minimise the potential sampling bias resulting from an oversampling of pregnant females when collecting “anonymous” faeces.

## Figures and Tables

**Table 1:** Variances in the number of pregnant females found in the early breeding period due to weather conditions from 2014 to 2019.

Parameters	2014	2015	2016	2017	2018	2019
Number of females with 20a-OHP levels ≥ 3000 ng/g	0	1	4	2	0	1
Number of females with 20α-OHP levels 2000–2999 ng/g	0	0	1	0	0	0
Number of females with 20α-OHP levels 1000–1999 ng/g	1	0	0	0	1	0
Number of females with 20α-OHP levels < 1000 ng/g	11	3	2	7	4	7
Snow height (mm) in January	132	48	35	50	130	84
Snow height (mm) in February	136	66	70	62	114	89
Snow height (mm) in March	93	60	61	21	124	88
Number of days with snowfall in January	13	13	5	15	11	5
Number of days with snowfall in February	11	14	8	5	6	9
Number of days with snowfall in March	9	10	5	12	9	11
Average daily temperature (°C) in January	–7.2	–7.9	–8.5	–11.9	–6.7	–11.3
Average daily temperature (°C) in February	–6.0	–8.9	–5.1	–5.2	–11.3	–6.5
Average daily temperature (°C) in March	–2.8	–2.8	–5.1	–0.8	–4.6	–3.6
Number of days with air frost in January	31	31	31	31	31	31
Number of days with air frost in February	28	28	29	28	28	28
Number of days with air frost in March	31	31	31	29	31	31

## Data Availability

All relevant data are within the paper.
